# [Retracted] Consecutive stimulation of HBsAg promotes the viability of the human B lymphoblastoid cell line IM‑9 through regulating the SIRT1‑NF‑κB pathway

**DOI:** 10.3892/ol.2023.13825

**Published:** 2023-04-19

**Authors:** Jian Bo, Xiaojuan Wang, Jie Li, Wenqing Wang, Jinqian Zhang

Oncol Lett 14: 433–440, 2017; DOI: 10.3892/ol.2017.6114

Following the publication of this paper, it was drawn to the Editor's attention by a concerned reader that the histone-3 and β-actin western blot data shown in Fig..3 were strikingly similar, albeit the bands appeared to have been placed into the figure in different orientations; in addition, certain of the western blotting data shown in Fig. 4 were strikingly similar to data that had appeared in different form in different articles published by (largely) different authors.

Owing to the fact that some of the data in the above article had already been published prior to its submission to *Oncology Letters*, the Editor has decided that this paper should be retracted from the Journal. The authors were asked for an explanation to account for these concerns, but the Editorial Office did not receive a satisfactory reply. The Editor apologizes to the readership for any inconvenience caused.

